# Novel Genetic and Phenotypic Expansion in *GOSR2*-Related Progressive Myoclonus Epilepsy

**DOI:** 10.3390/genes14101860

**Published:** 2023-09-25

**Authors:** Lea Hentrich, Mered Parnes, Timothy Edward Lotze, Rohini Coorg, Tom J. de Koning, Kha M. Nguyen, Calvin K. Yip, Heinz Jungbluth, Anne Koy, Hormos Salimi Dafsari

**Affiliations:** 1 Department of Pediatrics, Faculty of Medicine and University Hospital Cologne, University of Cologne, 50937 Cologne, Germany; lhentrich@age.mpg.de (L.H.);; 2 Max-Planck-Institute for Biology of Ageing, 50931 Cologne, Germany; 3 Cologne Excellence Cluster on Cellular Stress Responses in Aging Associated Diseases (CECAD), 50931 Cologne, Germany; 4 Division of Pediatric Neurology and Developmental Neuroscience, Department of Pediatrics, Baylor College of Medicine, Houston, TX 77030, USA; parnes@bcm.edu (M.P.); tlotze@bcm.edu (T.E.L.);; 5 Department of Genetics, University Medical Center Groningen, University of Groningen, 9713 GZ Groningen, The Netherlands; 6 Pediatrics, Department of Clinical Sciences, Lund University, 221 00 Lund, Sweden; 7 Department of Biochemistry and Molecular Biology, The University of British Columbia, Vancouver, BC V6T 1Z4, Canada; nguyenkm@student.ubc.ca (K.M.N.); calvin.yip@ubc.ca (C.K.Y.); 8 Department of Paediatric Neurology, Evelina’s Children Hospital, Guy’s & St. Thomas’ Hospital NHS Foundation Trust, London SE1 7EH, UK; 9 Randall Division of Cell and Molecular Biophysics, Muscle Signaling Section, King’s College London, London WC2R 2LS, UK; 10 Center for Rare Diseases, Faculty of Medicine and University Hospital Cologne, University of Cologne, 50937 Cologne, Germany

**Keywords:** *GOSR2*, North Sea progressive myoclonus epilepsy, vesicular trafficking, dystonia, clonazepam

## Abstract

Biallelic variants in the Golgi SNAP receptor complex member 2 gene (*GOSR2*) have been reported in progressive myoclonus epilepsy with neurodegeneration. Typical clinical features include ataxia and areflexia during early childhood, followed by seizures, scoliosis, dysarthria, and myoclonus. Here, we report two novel patients from unrelated families with a *GOSR2*-related disorder and novel genetic and clinical findings. The first patient, a male compound heterozygous for the *GOSR2* splice site variant c.336+1G>A and the novel c.364G>A,p.Glu122Lys missense variant showed global developmental delay and seizures at the age of 2 years, followed by myoclonus at the age of 8 years with partial response to clonazepam. The second patient, a female homozygous for the *GOSR2* founder variant p.Gly144Trp, showed only mild fine motor developmental delay and generalized tonic–clonic seizures triggered by infections during adolescence, with seizure remission on levetiracetam. The associated movement disorder progressed atypically slowly during adolescence compared to its usual speed, from initial intention tremor and myoclonus to ataxia, hyporeflexia, dysmetria, and dystonia. These findings expand the genotype–phenotype spectrum of *GOSR2*-related disorders and suggest that *GOSR2* should be included in the consideration of monogenetic causes of dystonia, global developmental delay, and seizures.

## 1. Introduction

Biallelic variants in the Golgi SNAP receptor complex member 2 gene (*GOSR2)* have been reported in progressive myoclonus epilepsy (PME, OMIM#614018). Patients usually present with ataxia and areflexia during early childhood, typically followed by seizures and myoclonus of upper limbs during the first decade of life [1]. Dysarthria, creatine kinase elevation, and scoliosis suggestive of a neuromuscular component are also common. Due to common ancestry of patients with a homozygous variant in NM_001012511.3:c.430G>T,p.Gly144Trp in countries in northwestern Europe, the condition was previously termed ‘North Sea’ PME (NS-PME). Cognitive function is typically spared at disease onset and may be followed by cognitive decline in adulthood. Fever and illness appear to irreversibly worsen NS-PME-associated symptoms, with limited recovery [2]. There are only supportive therapeutic options. *GOSR2* encodes the protein GOSR2/membrin that is involved in intracellular trafficking of COPII vesicles from the endoplasmic reticulum to cis-Golgi [3]. *GOSR2* loss-of-function variants prevent the membrane protein from localizing to cis-Golgi [4], as demonstrated in relevant animal models that demonstrate putative membrin loss of function in neurons and glia [5]. These animal models show similar phenotypes with seizures, particularly if the loss of function affects glial cell localization. In neurons, phenotype alignments are not observed, hinting at an involvement of glia in the pathophysiology of NSPME. However, the precise pathomechanisms and metabolic dysregulation of *GOSR2*-associated disorders remain largely elusive. Here, we report two novel patients with *GOSR2*-related myoclonus epilepsy with an expansion in genotype and phenotype in this ultra-rare disorder.

## 2. Materials and Methods

### 2.1. Patient Recruitment

The study was approved by local institutional review boards [6]. All patients and/or their legal guardians gave informed consent, including consent to the anonymous publication of their clinical information. Additional written informed consent was obtained from all participants (and/or their legal guardian) with identifiable photographs.

### 2.2. Molecular Genetic Investigations

We performed massively parallel sequencing with sequence alignment and variant calling as previously published [7,8]. We performed filtering for rare pathogenic variants below an allele frequency of <0.1% following recessive and dominant inheritance models. In a first step, we filtered for dystonia-associated genes (*GCH1, GCE, TH, SPR, ATP1A3, FTL, PRKRA, TUBB4A, BCAP31, COX20, KIF1C, SLC30A10, ANO3*) [7,9,10].

We performed further in silico and genomic analysis for all variants [11,12], including allele frequency in healthy population databases (gnomAD [13]), and pathogenicity prediction tools such as SpliceAI, GERP++, CADD-Phred, and MutPred2 [14,15,16,17]. Investigation of evolutionary conservation was based on NCBI HomoloGene sequences (Supplementary Table S2). All variants were scored on the basis of classification by the standards and guidelines of the American College of Medical Genetics and Genomics—American College of Molecular Pathology (ACMG) for the interpretation of variants [18].

To generate the structural model of the GOSR2/syntaxin-5/Bet1/Sec22b trans-SNARE complex, we used ColabFold and more specifically the AlphaFold-Multimer algorithm with the following protein sequences as input: GOSR2 (107–212), Syntaxin-5 (251–355), Bet1 (1–118), and Sec22b (105–215) [19]. The top-ranked model was subject to energy minimization using PHENIX [20]. The models of the mutants were generated using Pymol mutagenesis function. Figures of the trans-SNARE complex were prepared using Pymol.

## 3. Results

### 3.1. Phenotype Expansion

Patient 1 is a male from non-consanguineous Hispanic parents without any notable pregnancy or perinatal history. He showed a mild resting tremor in his hands shortly after falling while running at 18 months of age. On neurological examination at the age of two years his global development was delayed and he presented atypical absence epilepsy with myoclonic jerks exacerbated during a febrile illness. Initial neuroimaging was normal. EEG recordings showed bursts of generalized spike and slow wave activity of 2.5–3.5 Hz lasting up to 5 s. 76 seizures could be recorded within 24 h by EEG monitoring. He developed recurrent atonic seizures with falls at six years of age, and a movement disorder comprising multifocal myoclonus at eight years of age. At 12 years of age, the movement disorder progressed with ataxia after a generalized tonic-clonic status epilepticus. Anti-epileptic trials with lamotrigine, zonisamide, clobazam, ethosuximide, and topiramate were ineffective. Valproic acid led to remarkable improvements with seizure reduction to 1–2 per day, lasting 5–10 s, without falls. However, the family reported weakness after long walks in the context of valproic acid. The treatment was ultimately changed to levetiracetam which provided some clinical benefit. Clonazepam led to a partial improvement of the non-epileptic myoclonus. Laboratory investigations revealed an elevated creatine kinase (CK) level of 2900 IU/L. Muscle biopsy showed a mild increase in the number of mitochondria. At the most recent clinical assessment at 15 years of age, he showed mild intellectual disability, walked independently but was unsteady due to a broad based gait, and required assistance due to risk of falling. Current comorbidities include an attention deficit disorder, asthma, obstructive and central sleep apnea, and dysphagia.

**Figure 1:**
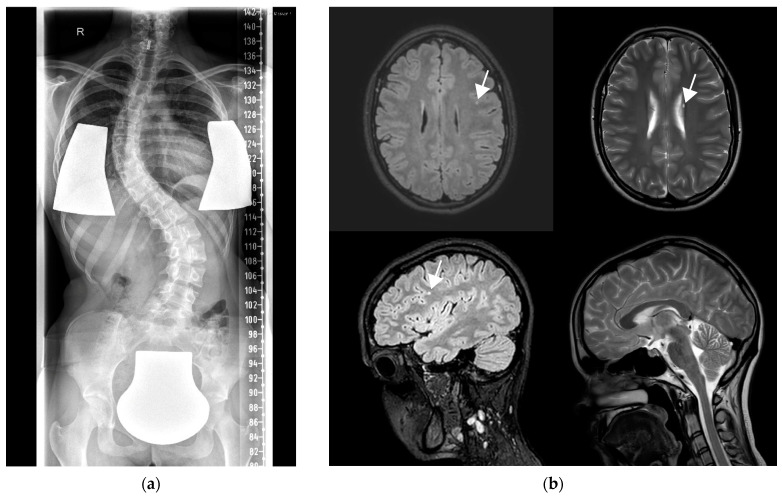
(**a**) X-ray showing scoliosis in patient 2 at the age of 17 years. (**b**) Brain MRI in patient 2 at the age of 17 years with left frontal FLAIR hyperintensity (upper left axial and lower left sagittal images), periventricular cyst (upper right axial) but no other abnormalities (lower right sagittal).

Patient 2 is female patient, who was born to non-consanguineous parents of German origin without any notable pregnancy or perinatal history. She presented with fine motor developmental delay with difficulties in handwriting at school age. At 12 years, she developed a generalized tonic–clonic seizure with photosensitivity after a viral infection triggered by flickering light, followed by permanent symptoms of intention tremor and shooting multifocal myoclonus. The movement disorder progressed gradually with increasing ataxia, dysmetria, and dystonia in the hands (Video S1). Hyporeflexia in the upper and lower limbs and scoliosis were noted on examination (Figure 1a). Each generalized tonic-–clonic seizure, three in total, led to a further deterioration of the movement disorder. After a urinary tract infection at the age of 17 years, the patient had a focal-onset atonic seizure affecting the lower limb. Interictal EEG recordings revealed multifocal generalized spike and sharp waves with bilateral frontal and occipital accentuation with secondary generalization without any clinical seizure correlations. Brain MRI studies showed left frontal FLAIR hyperintensities and a periventricular cyst (Figure 1b). Serum laboratory analyses revealed a persistent moderate CK elevation (500–1200 IU/L). Tandem mass spectrometry investigations for a range of metabolic diseases were unremarkable. Nerve conduction studies showed reduced sensory conduction velocity of the tibial nerve. An anti-epileptic trial with topiramate was unsuccessful but levetiracetam led to complete seizure remission. Trials with pyrimidone and propranolol did not have any effect on myoclonus and tremor, while carbamazepine only led to a brief improvement in myoclonus. At last follow-up at 20 years of age, the patient was seizure-free but wheelchair-bound. She did not show any signs of cognitive deterioration. The myoclonus of the limbs persisted on voluntary movements. She was unable to write and used a voice-guided computer. The family declined deep brain stimulation.

### 3.2. Molecular Genetic Investigations

**Figure 2:**
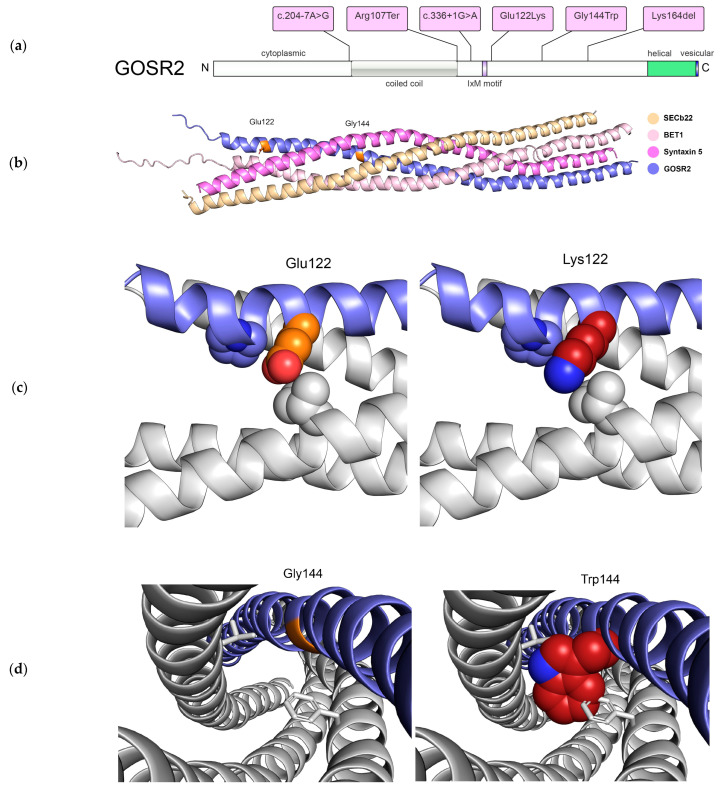
(**a**) Protein overview with all published variants including the p.Gly144Trp variant identified in patient 2 and the novel variants from patient 1 in c.336+1G>A and p.Glu122Lys. (**b**) Energy-minimized AlphaFold model of the SNARE complex composed of SEC22b (105–215), BET1 (1–118), Syntaxin-5 (251–355), and GOSR2 (107–212) colored in orange, pink, lavender, and blue, respectively. The locations of Glu122 and Gly144 are highlighted in orange. (**c**) Zoomed-in view of the structural models of wildtype complex and (left) complex containing GOSR2 p.Glu122Lys mutant (right) with the Glu122 residue colored in orange and Lys122 variant colored in red. These models suggest that the p.Glu122Lys mutation would have minimal impact on the SNARE assembly because different rotamers of Lys can be accommodated in this location. (**d**) Zoomed-in view of the structural models of wildtype complex and (left) complex containing GOSR2 p.Gly144Trp mutant (right) with the Gly144 residue colored in orange and Trp144 variant colored in red. These models show that the p.Gly144Trp mutation introduces steric hindrance with neighboring side chains and potentially destabilizes the SNARE complex assembly.

In patient 1, massively parallel sequencing revealed two novel compound heterozygous variants in *GOSR2*: NM_001012511.3:c.336+1G>A and c.364G>A,p.Glu122Lys. Analyses with SpliceAI showed a delta score at 0.99 for donor loss, predicting deficient protein expression. For the missense variant at p.Glu122Lys, the pathogenicity prediction tools showed a GERP score of 4.87, CADD-Phred score of 23.2, and allele count of 1/152204 in the gnomAD healthy population database [21]. MutPred2 analyses showed a score of 0.951 (loss of N-terminal acetylation *p* < 0.01, altered transmembrane protein *p* < 0.05). The p.Glu122Lys variant has previously been reported as a variant of unclear significance (VUS) with four submissions in ClinVar (ID 589948). On the basis of our clinical, genetic and bioinformatic analyses, we reclassified the variant as likely pathogenic (PM2, PM3, PP3, PP4). Protein models suggest that the p.Glu122Lys mutation would have minimal impact on the SNARE assembly because different rotamers of Lys can be accommodated in this location (Figure 2b,c).

In patient 2, karyotyping revealed 47,XXX without any relevance to the movement disorder. Array CGH did not detect pathogenic copy number alterations. Molecular genetic analysis via panel-based sequencing identified the pathogenic homozygous founder variant in *GOSR2* NM_001012511.3:c.430G>T,p.Gly144Trp, which was confirmed by Sanger sequencing. Pathogenicity prediction tools showed a CADD-Phred score of 32 and a MutPred2 score of 0.766 (altered disordered interface *p* < 0.05, altered transmembrane protein *p* < 0.01). Protein models showed that the p.Gly144Trp mutation introduces steric hindrance with neighboring side chains and potentially destabilizes the SNARE complex assembly (Figure 2b,d).

## 4. Discussion

PME is mainly characterized by seizures, movement disorders, and progressive neurological decline. The comprehensive genotype–phenotype spectrum in this disorder remains elusive due to limited reports. In *GOSR2*-associated disorders, previous attempts at genotype–phenotype analyses suggested a correlation between residual protein expression and disease severity, ultimately proposing a disease spectrum between PME with missense variants at the milder end and congenital muscular dystrophy with splice site variants at the more severe end [22].

A recent review of 21 patients with *GOSR2*-associated disorders showed the phenotype in the frequently observed p.Gly144Trp founder variant [2], while milder phenotypes with compound heterozygous variants (c.430G>T,c.491_493delAGA) [23] and three patients with α-dystroglycan muscular dystrophy showed a combination of the founder variant and splice site or missense variant that led to an effect in the N-terminal part of the protein (c.430G>T,c.336+1G>A; c.430G>T,c.2T>G; c.430G>T,c.82C>T) [22,24]. Table 1 demonstrates an overview of the core clinical features of *GOSR2*-related disorders in patients from our study and previously published literature. Supplementary Table S1 illustrates additional detailed reports of orthopedic findings and anti-epileptic trials in patients.

GOSR2/membrin is a member of the SNARE (soluble NSF (N-ethylmaleimide-sensitive factor) attachment protein receptor) family of proteins that mediate membrane fusion processes involving the ER, the ER–Golgi intermediate compartment, and the Golgi by forming a complex with three other SNAREs (Syntaxin-5, Bet1, and Sec22b) [3,30]. COPII-coated vesicles form on the endoplasmic reticulum (ER) from three subunits (Sar1-GTP, Sec23/24, Sec13/31) to bud vesicles for transport to the Golgi complex [31]. Interaction between the IxM motif in GOSR2 and the subunit Sec24c/d was reported to serve as the primary mechanism for packaging GOSR2/membrin and syntaxin-5 into COPII-coated vesicles for vesicular trafficking under healthy conditions [32].

Through analyses of the variants in our two patients and all published patients, we noted that all of the variants are found in the cytoplasmic part of the protein, some of which are essential for interacting with the SNARE complex (Figure 2a). All of the patient missense and truncating variants are located in regions that are highly conserved throughout evolution (Supplementary Table S2). Inspection of the AlphaFold2-predicted structural model of GOSR2 showed that the two mutation sites map to solvent-accessible regions and do not appear to alter the overall structure of this protein. To assess the effects of the disease mutations in the context of the assembled SNARE complex, we first generated a structural model of this complex composed of only the SNARE domains of GOSR2, syntaxin-5, Bet1, and Sec22b using AlphaFold2, followed by energy minimization refinement, and then constructed the models of the mutant complexes (Figure 2b) [19,20]. According to these models, the variant p.Gly144Trp introduces a large hydrophobic side chain that would sterically repel other side chain residues engaged in the interior of the coiled coil and potentially destabilize the assembly (Figure 2d). On the other hand, multiple lysine rotamers can be accommodated at position 122, which is located in the more solvent-accessible region near the tip of assembly (Figure 2c). Both variants in patient 1, the novel missense variant p.Glu122Lys and splice site variant c.336+1G>A, are located adjacent to the IxM motif. The essential splice variant is predicted to result into donor loss potentially disrupting the IxM motif for packaging GOSR2/membrin and syntaxin-5 into COPII-coated vesicles.

While the splice site variant was previously reported in a patient with muscular dystrophy, our patient 1 presented with a PME phenotype and no muscular hypotonia as in patients with p.Gly144Trp variants without infantile muscular hypotonia. The elevated CK levels with a peak of 2900 IU/L are similar to reports of PME patients in the literature. While this does not automatically preclude the patient from having muscular dystrophy, the muscle biopsy showed only mild increase of mitochondria and there were no other phenotypic signs indicative of muscular dystrophy in this patient, e.g., ocular structural changes, progressive loss of muscle mass, or Gower’s sign [7,33]. Indeed, while elevated CK levels may prompt further investigations on future clinical presentation, any CK elevation here was interpreted as likely arising from myoclonus.

Our patient 2 with the founder variant p.Gly144Trp illustrates a milder and later onset of NS-PME with gradual progression of the movement disorder and without cognitive decline. The phenotype expansion included dystonia that may present in other patients with later onsets of movement disorders. While this is not the oldest reported patient to date, the movement disorders began with myoclonus at 13 years and ataxia at 16 years of age. The latest onsets of movement disorders from published reports were at 13 years for ataxia and eight years for myoclonus. Complete seizure remission on levetiracetam monotherapy as in our patient 2 was previously reported in a patient with biallelic *TRAPPC11* variants and congenital muscular dystrophy. TRAPPC11 and GOSR2 are both involved in COPII-related trafficking [34]. Recent work indicated that TRAPPC11 recruits ATG2B–WDR45 to pre-autophagosomal membranes and its Atg9-mediated autophagosome closure during macroautophagy [35], while WDR45 facilitates autophagolysosomal fusion by interacting with the tethering factor EPG5 in neural cells [36]. Monogenic disorders in this pathway include WDR45 deficiency with a bi-phasic disorder from developmental and epileptic–dyskinetic encephalopathy to adult dystonia–Parkinsonism [37]. EPG5 deficiency is associated with a wide spectrum from neurodevelopmental disorders and seizures to Vici syndrome with multi-system features including neurodevelopmental disorders, movement disorders, epilepsy, and immunodeficiency [38,39]. Several anti-epileptic drugs have been included in the management of these disorders of autophagy and intracellular trafficking that may modulate exocytosis or synaptic trafficking, e.g., gabapentin, carbamazepine, or clonazepam [40]. Recent reports of spatial proteomics in *TECPR2*-related hereditary sensory and autonomic neuropathy indeed also implicated these vesicular trafficking pathways, specifically ER-to-Golgi transport via coated vesicles [41].

Notably, phenotype progression in patient 2 occurred on repeated viral infections. It remains elusive how viral infections trigger the disease progression. Recent findings have implicated a viral hijacking of COPII-coated vesicles including flaviviruses and SARS-CoV-2 [42,43]. These viral hijacking routes may be of specific interest as they show a pathomechanism related to COPII vesicle trafficking. For instance, the hepatitis C virus is budded in the endoplasmic reticulum lumen and exits via COPII vesicles by modulating the protein milieu and reorganization at organellar sites such as ER–Golgi intermediate compartments [44]. Future studies may address how viral infection potentially adds on to disorders in COPII-related trafficking or autophagosome biogenesis on the background of biallelic GOSR2 deficiency.

Another important factor in the consideration of viral infections as triggers for disease progression is heat insensitivity itself as it has been reported that patients with GOSR2 deficiency are sensitive to all forms of heat-inducing stressors [5], perhaps indicating a more general mechanism of protein misfolding and ER stress in *GOSR2*-related disorders. Indeed, a *Drosophila melanogaster* model with knockdown of *GOSR2* ortholog membrin indicated seizure-like behavior with hyperthermia. Reduced membrin expression in glia rendered the flies particularly prone to age-dependent seizures. Of note, there was a partial response to treatment with sodium barbital in flies. However, this was not trialed in our patients due to its adverse effects.

It remains unclear which viral pathogens led to the progression in our patient. The additional report of a generalized tonic–clonic seizure after a urinary tract infection in our patient may also suggest a role of bacterial pathogens in disease progression. Recent reports implicated *Legionella pneumophila* in subverting functions of Rab1 and Sec22b for its replication in cells [45]. As Sec24 phosphorylation regulates autophagosome abundance during nutrient deprivation by interacting with Atg9, COPII-related trafficking serves an important mechanism for autophagosome formation [46]. Recently, biallelic variants in *ATG9A* were reported in immune dysregulation with diplopia and inflammatory lesions in brain MRI studies after EBV infection [47]. Other innate errors of autophagy and intracellular trafficking with neurological symptoms after recurrent infections include disorders in phosphatidylinositol 4-kinases which are recruited by the membrin–Arf1 complex in vesicular trafficking [6,48]. Further work is needed to elucidate the role of viral and bacterial infections in the disease progression in patients with GOSR2 deficiency and how pathogen-induced perturbation of vesicular trafficking may eventually trigger neurodegeneration.

## 5. Conclusions

In conclusion, we report a genotypic and phenotypic expansion of *GOSR2*-associated NS-PME in a patient with early-onset disease and refractory epilepsy likely due to disrupted interaction with the COPII-coated vesicle machinery, and another patient with disease onset during adolescence, seizure remission on levetiracetam, and retained cognitive function during the third decade of life. The presentation with juvenile-onset dystonia suggests that *GOSR2* may also be included in the differential diagnoses of monogenetic dystonia. 

## Figures and Tables

**Table 1:** Overview on core features of patients from this study and the published literature. Abbreviations: y = years; m = months; w = weeks; hom = homozygous; comp het = compound heterozygous; + = present; − = not present; ND = not defined; GTCS = generalized tonic–clonic seizures; ID = intellectual disability; CD = cognitive decline; LD = learning disability; DBS = deep brain stimulation.

Study	Case No. (Age Last Reported)	Genotype—Inheritance Mode, cDNA NM_001012511.3 (Protein NP_004278.2)	Ataxia (Age at Onset)	Myoclonus (Age at Onset)	Seizures (Age at Onset)	Others
Corbett et al., 2011 [1]; Boissé Lomax et al., 2013 [25]	#01 (32 y)	hom, c.430G>T (p.Gly144Trp)	+ (2 y)	+ (8 y)	+ (absence 7y, GTCS with 13 y, drop attacks 13 y)	CD (25 y); pneumonia
	#02 (17 y)	hom, c.430G>T (p.Gly144Trp)	+ (1 y)	+ (6 y)	+ (drop attacks 13 y)	ID
	#03 (32 y)	hom, c.430G>T (p.Gly144Trp)	+ (3 y)	+ (6 y)	+ (GTCS 14 y)	
	#04 (30 y)	hom, c.430G>T (p.Gly144Trp)	+ (2 y)	+ (10 y)	+ (absence 6 y, GTCS 12 y, drop attacks 14 y)	CD (25 y)
	#05 (24 y)	hom, c.430G>T (p.Gly144Trp)	+ (2 y)	+ (6 y)	+ (GTCS 21 y)	CD (30 y)
	#06 (29 y)	hom, c.430G>T (p.Gly144Trp)	+ (2 y)	+ (5 y)	+ (GTCS 24 y, absence 24 y)	
Boissé Lomax et al., 2013 [25]	#07 (10 y)	hom, c.430G>T (p.Gly144Trp)	+ (1 y)	+ (4.5 y)	+ (focal seizures 14 m, GTCS 8.5 y)	
	#08 (29 y)	hom, c.430G>T (p.Gly144Trp)	+ (3 y)	+ (5 y)	+ (drop attacks 2 y, absence 2 y, GTCS 21 y)	LD; febrile seizures
	#09 (27 y)	hom, c.430G>T (p.Gly144Trp)	+ (3.5 y)	+ (4 y)	+ (absence 5 y, GTCS 8 y)	
	#10 (18 y)	hom, c.430G>T (p.Gly144Trp)	+ (2 y)	+ (6 y)	+ (GTCS 10 y, absence)	
	#11 (37 y)	hom, c.430G>T (p.Gly144Trp)	+ (3 y)	+ (12 y)	+ (absence 3 y, GTCS 3 y)	
	#12 (13 y)	hom, c.430G>T (p.Gly144Trp)	+ (2.3 y)	+ (6 y)	+ (GTCS 12 y)	
Tsai et al., 2013 [22]	#13 (36 w)	comp het, c.430G>T (p.Gly144Trp), c.336+1G>A	−	−	−	Muscular dystrophy
van Egmond et al., 2014 [26]	#14 (19 y)	hom, c.430G>T (p.Gly144Trp)	+ (3 y)	+ (5 y)	+ (tonic seizures 9 y)	
	#15 (26 y)	hom, c.430G>T (p.Gly144Trp)	+ (3 y)	+ (6 y)	+ (GTCS 11 y)	Mild LD
	#16 (20 y)	hom, c.430G>T (p.Gly144Trp)	+ (2 y)	+ (6 y)	+ (GTCS 6 y)	Mild LD
	#17 (12 y)	hom, c.430G>T (p.Gly144Trp)	+ (5 y)	+ (8 y)	+ (clonic seizures 3 y)	
	#18 (7 y)	hom, c.430G>T (p.Gly144Trp)	+ (3 y)	+ (6 y)	NA	
Praschberger et al., 2015 [23]	#19 (61 y)	comp het, c.430G>T (p.Gly144Trp), c.491_493delAGA (p.Lys164del)	+ (2 y)	+ (14 y)	+ (14 y)	Mild CD
Anderson et al., 2016 [27]	#20 (10 y)	hom, c.430G>T (p.Gly144Trp)	+ (ND)	+ (5 y)	+ (GTCS 6 y)	DBS
	#21 (30 y)	hom, c.430G>T (p.Gly144Trp)	+ (ND)	+ (ND)	+ (GTCS 8 y)	DBS
	#22 (27 y)	ND	+ (ND)	+ (3 y)	+ (GTCS 5 y)	DBS
Larson et al., 2018 [28]	#23 (died at 5 y)	ND	−	−	+ (absence 2 y)	Muscular dystrophy
	#24 (6 y)	comp het, c.430G>T (p.Gly144Trp), c.2T>G (p.Met1Arg)	−	−	+ (2.5 y)	Muscular dystrophy
Polet et al., 2020 [2]	#25 (5 y)	hom, c.430G>T (p.Gly144Trp)	+ (4 y)	+ (4 y)	NA	
	#26 (8 y)	hom, c.430G>T (p.Gly144Trp)	+ (2 y)	+ (5 y)	+ (7 y)	
	#27 (8 y)	hom, c.430G>T (p.Gly144Trp)	+ (2 y)	+ (3 y)	+ (7 y)	
	#28 (13 y)	hom, c.430G>T (p.Gly144Trp)	+ (3 y)	+ (6 y)	NA	
	#29 (18 y)	hom, c.430G>T (p.Gly144Trp)	+ (5 y)	+ (7 y)	+ (3 y)	DBS
	#30 (19 y)	hom, c.430G>T (p.Gly144Trp)	+ (4 y)	+ (7 y)	+ (9 y)	DBS
	#31 (20 y)	hom, c.430G>T (p.Gly144Trp)	+ (5 y)	+ (5 y)	+ (13 y)	
	#32 (26 y)	hom, c.430G>T (p.Gly144Trp)	+ (3 y)	+ (5 y)	+ (9 y)	
	#33 (26 y)	hom, c.430G>T (p.Gly144Trp)	+ (2 y)	+ (6 y)	+ (8 y)	
	#34 (31 y)	hom, c.430G>T (p.Gly144Trp)	+ (5 y)	+ (8 y)	+ (8 y)	
	#35 (31 y)	hom, c.430G>T (p.Gly144Trp)	+ (8 y)	+ (9 y)	+ (8 y)	
	#36 (32 y)	hom, c.430G>T (p.Gly144Trp)	+ (3 y)	+ (6 y)	+ (11 y)	
	#37 (35 y)	hom, c.430G>T (p.Gly144Trp)	+ (5 y)	+ (ND)	+ (ND)	DBS
	#38 (36 y)	hom, c.430G>T (p.Gly144Trp)	+ (ND)	+ (ND)	+ (6 y)	
	#39 (37 y)	hom, c.430G>T (p.Gly144Trp)	+ (5 y)	+ (ND)	+ (8 y)	DBS
	#40 (41 y)	hom, c.430G>T (p.Gly144Trp)	+ (ND)	+ (5 y)	+ (8 y)	
	#41 (46 y)	hom, c.430G>T (p.Gly144Trp)	+ (3 y)	+ (4 y)	+ (6 y)	
Stemmerik et al., 2021 [29]	#42 (48 y)	comp het, c.319C>T (p.Arg107Ter), c.204-7A>G (p.?)	+ (13 y)	+ (7 y)	NA	Mild CD (46 y)
This study	#43 (15 y)	comp het, c.364G>A (p.Glu122Lys), c.336+1G>A (p.?)	+ (12 y)	+ (8 y)	+ (2 y)	Mild ID
	#44 (21 y)	hom, c.430G>T (p.Gly144Trp)	+ (16 y)	+ (13 y)	+ (GTCS 12y on urinary tract infection)	Gradual progression of movement disorders into dystonia on recurrent viral infections

## Data Availability

Anonymized data are available upon request from the corresponding author.

## Supplementary Materials

The following supporting information can be downloaded at https://www.mdpi.com/article/10.3390/genes14101860/s1: Video S1. Patient with *GOSR2*-associated North Sea progressive myoclonus epilepsy shows tremor and myoclonus of the upper limbs during arm holding test, finger tapping test, and spontaneous movements, at the age of 20 years. The patient shows dystonic posturing of the hands during the arm holding test and the finger tapping test; Table S1. Detailed overview on all patients with GOSR2-related disorders from the published literature and our two novel patients regarding publication, sex, age in years, genetic variant, protein mutation, variant zygosity, first symptoms (with age at onset), ataxia (with age at onset), myoclonus (with age at onset), seizures (with age at onset), orthopedic complications, cognition, mobility at last follow-up, highest recorded CK level (U/L), recurrent infections, management (successful in bold), and others. Abbreviations: LVT, levetiracetam; VPA, valproic acid; CLN, clonazepam; CBZ, clobazam; MDZ, midazolam; PCT, piracetam; PRP, perampanel; PYR: pyrimidone; PRO: propanolol; ESM, ethosuximide; ZNS, zonisamide; ATZ, acetazolamide; LMT, lamotrigine; FBM, felbamate; BCF, baclofen; CRN, carnitine; CBM, carbamazepine; MPD, methylpredisolon; 5HTP, 5-hydroxytryptofaan; CBI, carbidopa; PHB, phenobarbital; DBS, deep brain stimulation; Table S2. Wildtype protein and variants in *Homo sapiens*, as well as illustration of evolutionary conservation of residues with wildtype sequences in *Pan troglodytes*, *Macaca mulatta*, *Canis lupus*, *Bos taurus*, *Gallus gallus*, *Danio rerio*, *Drosophila melanogaster*, *Caenorhabditis elegans*, and *Xenopus tropicalis*.

## References

[1] Corbett M.A., Schwake M., Bahlo M., Dibbens L.M., Lin M., Gandolfo L.C., Vears D.F., O’Sullivan J.D., Robertson T., Bayly M.A. (2011). A Mutation in the Golgi Qb-SNARE Gene GOSR2 Causes Progressive Myoclonus Epilepsy with Early Ataxia. Am. J. Hum. Genet..

[2] Polet S.S., Anderson D.G., Koens L.H., van Egmond M.E., Drost G., Brusse E., Willemsen M.A., Sival D.A., Brouwer O.F., Kremer H.P. (2020). A detailed description of the phenotypic spectrum of North Sea Progressive Myoclonus Epilepsy in a large cohort of seventeen patients. Park. Relat. Disord..

[3] Lowe S.L., Peter F., Subramaniam V.N., Wong S.H., Hong W. (1997). A SNARE involved in protein transport through the Golgi apparatus. Nature.

[4] Dibbens L.M., Rubboli G. (2016). GOSR2: A progressive myoclonus epilepsy gene. Epileptic Disord..

[5] Lambrechts R.A., Polet S.S., Hernandez-Pichardo A., van Ninhuys L., Gorter J.A., Grzeschik N.A., de Koning-Tijssen M.A.J., de Koning T.J., Sibon O.C.M. (2019). North Sea Progressive Myoclonus Epilepsy is Exacerbated by Heat, A Phenotype Primarily Associated with Affected Glia. Neuroscience.

[6] Dafsari H.S., Pemberton J.G., Ferrer E.A., Yammine T., Farra C., Mohammadi M.H., Ghayoor Karimiani E., Hashemi N., Souaid M., Sabbagh S. (2022). PI4K2A deficiency causes innate error in intracellular trafficking with developmental and epileptic-dyskinetic encephalopathy. Ann. Clin. Transl. Neurol..

[7] Bayram N., Kaçar Bayram A., Daimagüler H.S., Dafsari H.S., Bamborschke D., Uyanik G., Erdogan M., Özsaygılı C., Pangal E., Yuvaci İ. (2021). Genotype-phenotype correlations in ocular manifestations of Marinesco-Sjögren syndrome: Case report and literature review. Eur. J. Ophthalmol..

[8] Dafsari H.S., Kawalia A., Sprute R., Karakaya M., Malenica A., Herkenrath P., Nürnberg P., Motameny S., Thiele H., Cirak S. (2019). Novel mutations in SLC6A5 with benign course in hyperekplexia. Cold Spring Harb. Mol. Case Stud..

[9] Dafsari H.S., Sprute R., Wunderlich G., Daimagüler H.-S., Karaca E., Contreras A., Becker K., Schulze-Rhonhof M., Kiening K., Karakulak T. (2019). Novel mutations in KMT2B offer pathophysiological insights into childhood-onset progressive dystonia. J. Hum. Genet..

[10] Saffari A., Lau T., Tajsharghi H., Karimiani E.G., Kariminejad A., Efthymiou S., Zifarelli G., Sultan T., Toosi M.B., Sedighzadeh S. (2023). The clinical and genetic spectrum of autosomal-recessive TOR1A-related disorders. Brain.

[11] Becker L.L., Dafsari H.S., Schallner J., Abdin D., Seifert M., Petit F., Smol T., Bok L., Rodan L., Krapels I. (2020). The clinical-phenotype continuum in DYNC1H1-related disorders—Genomic profiling and proposal for a novel classification. J. Hum. Genet..

[12] Sprute R., Jergas H., Ölmez A., Alawbathani S., Karasoy H., Dafsari H.S., Becker K., Daimagüler H.S., Nürnberg P., Muntoni F. (2021). Genotype-phenotype correlation in seven motor neuron disease families with novel ALS2 mutations. Am. J. Med. Genet. A.

[13] Karczewski K.J., Francioli L.C., Tiao G., Cummings B.B., Alföldi J., Wang Q., Collins R.L., Laricchia K.M., Ganna A., Birnbaum D.P. (2020). The mutational constraint spectrum quantified from variation in 141,456 humans. Nature.

[14] Pejaver V., Urresti J., Lugo-Martinez J., Pagel K.A., Lin G.N., Nam H.-J., Mort M., Cooper D.N., Sebat J., Iakoucheva L.M. (2020). Inferring the molecular and phenotypic impact of amino acid variants with MutPred2. Nat. Commun..

[15] Rentzsch P., Witten D., Cooper G.M., Shendure J., Kircher M. (2019). CADD: Predicting the deleteriousness of variants throughout the human genome. Nucleic Acids Res..

[16] de Sainte Agathe J.M., Filser M., Isidor B., Besnard T., Gueguen P., Perrin A., Van Goethem C., Verebi C., Masingue M., Rendu J. (2023). SpliceAI-visual: A free online tool to improve SpliceAI splicing variant interpretation. Hum. Genom..

[17] Davydov E.V., Goode D.L., Sirota M., Cooper G.M., Sidow A., Batzoglou S. (2010). Identifying a high fraction of the human genome to be under selective constraint using GERP++. PLoS Comput. Biol..

[18] Richards S., Aziz N., Bale S., Bick D., Das S., Gastier-Foster J., Grody W.W., Hegde M., Lyon E., Spector E. (2015). Standards and guidelines for the interpretation of sequence variants: A joint consensus recommendation of the American College of Medical Genetics and Genomics and the Association for Molecular Pathology. Genet. Med..

[19] Jumper J., Evans R., Pritzel A., Green T., Figurnov M., Ronneberger O., Tunyasuvunakool K., Bates R., Žídek A., Potapenko A. (2021). Highly accurate protein structure prediction with AlphaFold. Nature.

[20] Afonine P.V., Grosse-Kunstleve R.W., Echols N., Headd J.J., Moriarty N.W., Mustyakimov M., Terwilliger T.C., Urzhumtsev A., Zwart P.H., Adams P.D. (2012). Towards automated crystallographic structure refinement with phenix.refine. Acta Crystallogr. D Biol. Crystallogr..

[21] Dafsari H.S., Becker L., von der Hagen M., Cirak S. (2021). Genomic profiling in neuronal dyneinopathies and updated classifications. Am. J. Med. Genet. A.

[22] Tsai L., Schwake M., Corbett M.A., Gecz J., Berkovic S., Shieh P.B. (2013). P. 1.20 GOSR2: A novel form of Congenital Muscular Dystrophy. Neuromuscul. Disord..

[23] Praschberger R., Balint B., Mencacci N.E., Hersheson J., Rubio-Agusti I., Kullmann D.M., Bettencourt C., Bhatia K., Houlden H. (2015). Expanding the Phenotype and Genetic Defects Associated with the GOSR2 Gene. Mov. Disord. Clin. Pract..

[24] Henige H., Kaur S., Pappas K. (2021). Compound heterozygous variants in GOSR2 associated with congenital muscular dystrophy: A case report. Eur. J. Med. Genet..

[25] Boissé Lomax L., Bayly M.A., Hjalgrim H., Møller R.S., Vlaar A.M., Aaberg K.M., Marquardt I., Gandolfo L.C., Willemsen M., Kamsteeg E.J. (2013). “North Sea” Progressive Myoclonus Epilepsy: Phenotype of Subjects with GOSR2 Mutation. Brain.

[26] van Egmond M.E., Verschuuren-Bemelmans C.C., Nibbeling E.A., Elting J.W.J., Sival D.A., Brouwer O.F., de Vries J.J., Kremer H.P., Sinke R.J., Tijssen M.A. (2014). Ramsay Hunt Syndrome: Clinical Characterization of Progressive Myoclonus Ataxia Caused by GOSR2 Mutation. Mov. Disord..

[27] Anderson D.G., Németh A.H., Fawcett K.A., Sims D., Miller J., Krause A. (2016). Deep Brain Stimulation in Three Related Cases of North Sea Progressive Myoclonic Epilepsy from South Africa. Mov. Disord. Clin. Pract..

[28] Larson A.A., Baker P.R., Milev M.P., Press C.A., Sokol R.J., Cox M.O., Lekostaj J.K., Stence A.A., Bossler A.D., Mueller J.M. (2018). TRAPPC11 and GOSR2 Mutations Associate with Hypoglycosylation of α-Dystroglycan and Muscular Dystrophy. Skelet. Muscle.

[29] Stemmerik M.G., Borch J.d.S., Dunø M., Krag T., Vissing J. (2021). Myopathy Can Be a Key Phenotype of Membrin (GOSR2) Deficiency. Hum. Mutat..

[30] Hay J.C., Klumperman J., Oorschot V., Steegmaier M., Kuo C.S., Scheller R.H. (1998). Localization, dynamics, and protein interactions reveal distinct roles for ER and Golgi SNAREs. J. Cell Biol..

[31] Lee M.C.S., Miller E.A., Goldberg J., Orci L., Schekman R. (2004). Bi-directional protein transport between the ER and Golgi. Annu. Rev. Cell Dev. Biol..

[32] Mancias J.D., Goldberg J. (2008). Structural basis of cargo membrane protein discrimination by the human COPII coat machinery. EMBO J..

[33] Dafsari H.S., Kocaturk N.M., Daimagüler H.-S., Brunn A., Dötsch J., Weis J., Deckert M. (2019). Bi-allelic mutations in uncoordinated mutant number-45 myosin chaperone B are a cause for congenital myopathy. Acta Neuropathol. Commun..

[34] Zhao S., Li C.M., Luo X.M., Siu G.K., Gan W.J., Zhang L., Wu W.K., Chan H.C., Yu S. (2017). Mammalian TRAPPIII Complex positively modulates the recruitment of Sec13/31 onto COPII vesicles. Sci. Rep..

[35] Stanga D., Zhao Q., Milev M.P., Saint-Dic D., Jimenez-Mallebrera C., Sacher M. (2019). TRAPPC11 functions in autophagy by recruiting ATG2B-WIPI4/WDR45 to preautophagosomal membranes. Traffic.

[36] Ji C., Zhao H., Chen D., Zhang H., Zhao Y.G. (2021). β-propeller proteins WDR45 and WDR45B regulate autophagosome maturation into autolysosomes in neural cells. Curr. Biol..

[37] Saffari A., Schröter J., Garbade S.F., Alecu J.E., Ebrahimi-Fakhari D., Hoffmann G.F., Kölker S., Ries M., Syrbe S. (2022). Quantitative retrospective natural history modeling of WDR45-related developmental and epileptic encephalopathy—A systematic cross-sectional analysis of 160 published cases. Autophagy.

[38] Dafsari H.S., Ebrahimi-Fakhari D., Saffari A., Deneubourg C., Fanto M., Jungbluth H. (2022). EPG5-Related Disorder. http://www.ncbi.nlm.nih.gov/pubmed/29227033.

[39] Byrne S., Dionisi-Vici C., Smith L., Gautel M., Jungbluth H. (2016). Vici syndrome: A review. Orphanet J. Rare Dis..

[40] Allen N.M., Dafsari H.S., Wraige E., Jungbluth H. (2018). Neck-Tongue Syndrome: An Underrecognized Childhood Onset Cephalalgia. J. Child. Neurol..

[41] Nalbach K., Schifferer M., Bhattacharya D., Ho-Xuan H., Tseng W., Williams L.A., Stolz A., Lichtenthaler S.F., Elazar Z., Behrends C. (2023). Spatial proteomics reveals secretory pathway disturbances caused by neuropathy-associated TECPR2. Nat. Commun..

[42] Hassan Z., Kumar N.D., Reggiori F., Khan G. (2021). How Viruses Hijack and Modify the Secretory Transport Pathway. Cells.

[43] Cattin-Ortolá J., Welch L.G., Maslen S.L., Papa G., James L.C., Munro S. (2021). Sequences in the cytoplasmic tail of SARS-CoV-2 Spike facilitate expression at the cell surface and syncytia formation. Nat. Commun..

[44] Avula K., Singh B., Samantaray S., Syed G.H. (2023). The Early Secretory Pathway Is Crucial for Multiple Aspects of the Hepatitis C Virus Life Cycle. J. Virol..

[45] Kagan J.C., Stein M.P., Pypaert M., Roy C.R. (2004). Legionella Subvert the Functions of Rab1 and Sec22b to Create a Replicative Organelle. J. Exp. Med..

[46] Davis S., Wang J., Zhu M., Stahmer K., Lakshminarayan R., Ghassemian M., Jiang Y., Miller E.A., Ferro-Novick S. (2016). Sec24 phosphorylation regulates autophagosome abundance during nutrient deprivation. eLife.

[47] Hu G., Hauk P.J., Zhang N., Elsegeiny W., Guardia C.M., Kullas A., Crosby K., Deterding R.R., Schedel M., Reynolds P. (2023). Autophagy-associated immune dysregulation and hyperplasia in a patient with compound heterozygous mutations in ATG9A. Autophagy.

[48] De Matteis M.A., Godi A. (2004). PI-loting membrane traffic. Nat. Cell Biol..

